# Elucidation of flavor codes in citrus fruit peels using electronic sensors, LC-MS/MS, and GC–MS combined with GC-O

**DOI:** 10.1016/j.fochx.2026.104074

**Published:** 2026-06-06

**Authors:** Hee Sung Moon, Seong Jun Hong, Se Young Yu, Hyeonjin Park, Younglan Ban, Gwang-Ju Jang, Ho Eun Kim, Sang-Hee Lee, Eui-Cheol Shin

**Affiliations:** a Department of GreenBio Science (BK21)/Food Science and Technology, Gyeongsang National University, Jinju 52828, Republic of Korea; b Korea Food Research Institute, Wanju-gun 55365, Republic of Korea; c Department of Food Biotechnology, University of Science and Technology, Daejeon 34113, Republic of Korea

**Keywords:** Citrus peels, Byproduct, *E*-sensing techniques, Molecular structure, Flavor code

## Abstract

This study integrated biomimetic sensors with targeted taste compounds (organic acids; OAs and free sugars; FSs) and odor compounds profiling GC–MS-O to characterize citrus fruit peels (CFP) across seven cultivars. Among FSs, fructose and glucose were predominant (range: 25.68–8.16 mg/g and 24.11–7.51 mg/g, respectively), while sucrose was highest in kumquat peel (KP) (18.48 ± 0.19 mg/g). Citric acid and quinic acid were the major OAs, with citric acid peaking in KP (2152.38 ± 18.84 mg/kg) and quinic acid in lemon peel (LP) (1018.75 ± 15.90 mg/kg) (*p* < 0.05). *E*-tongue profiles revealed clear cultivar-dependent separation along [sourness/sweetness/umami] dimensions, consistent with the acid–sugar balance. Odor compounds analysis identified 60 compounds, and GC–O/odor-active value (OAV) highlighted odor compounds (decanal, *α*-pinene, limonene, 2-carene, and *γ*-muurolene) as key odor contributors (OAV > 1). Multivariate integration linked sensor outputs to metabolite signatures, providing an evidence-based framework to prioritize CFP types for targeted flavor applications.

## Highlights

• The flavor of citrus fruit peels was analyzed by electronic sensors and chromatographic methods.
• The organic acids and free sugars were analyzed using LC-MS/MS and GC–MS.
• Multivariate analysis was conducted based on the 52 metabolites of CFPs identified.
• Citrus fruit peel flavor metabolites positively correlated with sourness.
• Citrus fruit peel flavor metabolites negatively correlated with bitterness and sweetness.

## 1 Introduction

Citrus fruits (CFs) are among the most widely consumed fruits, including orange (*Citrus sinensis*), mandarin orange (*C. reticulata*), and lemon (*C. limon*) (Sanli et al., 2025). Citrus fruits provide consumers with substantial nutritional value and health benefits due to their high content of secondary metabolites, and the annual production of citrus fruits is approximately 98.7 million tons (Lee et al., 2024; Perez-Rosas et al., 2026). Generally, the pulp of citrus fruits is primarily used for juice production. The waste generated after juice processing, including citrus fruit peel (CFP), albedo, and seeds (approximately 45% of the total fruit weight), is considered agricultural waste, resulting in about 40 million tons of byproduct (Perez-Rosas et al., 2026; Sanli et al., 2025). These byproducts are often discarded in landfills or marine environments, causing serious environmental concerns and high disposal costs (Lee et al., 2014; Lee et al., 2024; Wang et al., 2023). CFPs, which are considered byproducts, exhibit antioxidant and anti-inflammatory activities and are known to be rich in odor compounds (Huang & Ho, 2010; Zhang et al., 2017). Typically, CFPs contain predominant limonoids, phenolic acids, odor compounds, and fiber in greater amounts compared with the pulps (Huang & Ho, 2010). For these reasons, CFPs possess notable industrial potential in the food industry.

Recently, various studies on the organoleptic properties of foods have been reported, leading to increased interest in the flavor profile of plants and fruits (Gómez-Mejía et al., 2025; Jo et al., 2025). Various analytical systems are now widely employed to investigate the flavor profiles of CFs (Jo et al., 2025; Wang et al., 2023). For instance, LC-MS/MS, electronic sensors including the electronic tongue (*E*-tongue) and electronic nose (E-nose), and GC–MS are commonly employed to analyze odor compounds and flavor patterns in foods (Jo et al., 2025; Li et al., 2022; Tan & Xu, 2020). The integrated utilization of these analytical systems facilitates the identification of flavor metabolites that influence human flavor perception, directly and indirectly.

Despite this methodological progress, CFP flavor characterization still faces two practical gaps. First, cultivar-dependent differences in taste- and odor-relevant chemistry—particularly organic acids (OAs), free sugars (FSs), and odor-active compounds (OACs), are often studied in a limited number of cultivars or under heterogeneous experimental conditions, restricting cross-cultivar comparability (Asencio et al., 2018; Jo et al., 2025; Ren et al., 2015). Second, many studies focus on either odor or taste fractions alone, whereas product-relevant flavor perception and industrial suitability are shaped by the joint contributions and interactions of taste-active and odor-active components. Therefore, the present study investigated the integrated flavor attributes of seven CFPs. We combined electronic sensing (*E*-tongue/E-nose) with cross-platform chemical analysis (LC–MS/MS and GC–O–MS) and quantified key contributors using contribution metrics (taste-active value, TAV; odor-active value, OAV). Multivariate and network-based integration was further applied to identify cultivar-specific “flavor metabolite signatures” and to propose a practical screening framework for CFP selection. We hypothesized that (i) cultivar-dependent variation in the OA–FS matrix would be reflected in distinct E-tongue taste dimensions (e.g., sourness-related vs sweetness-related signals), and (ii) a limited number of OACs would account for cultivar-specific aroma signatures observed by GC–O and E-nose patterns. We further hypothesized that contribution metrics (TAV/OAV and TC/OC) combined with multivariate analyses (PCA/PLS-based discrimination and correlation/network approaches) would enable an integrated, correlation-based ‘flavor-metabolite fingerprint’ across citrus peels. Collectively, this study is positioned as an application-oriented screening and interpretation study rather than a receptor-level mechanistic investigation. If future studies combine receptor-based analyses with human sensory validation, it will be possible to further solidify the mechanistic understanding of the key candidate biomarkers identified in this study.

## 2 Materials and methods

### 2.1 Material preparation

Cultivars were selected to represent both genetically and phenotypically distinct citrus species and commercially important mandarin hybrid cultivars, covering a wide range of flavor-relevant metabolite diversity. The CF varieties used in this experiment comprised orange (*Citrus sinensis*, Country of Origin: California, USA) peel (OP), mandarin orange (*C. reticulata*, Country of Origin: Jeju-island, Republic of Korea) peel (MP), lemon (*C. limon*, Country of Origin: California, USA) peel (LP), cheonhyehyang (*C. setoka*; *C. kiyomi* × *C. encore*, Country of Origin: Jeju-island, Republic of Korea) peel (CP), redhyang (*C. kanpei*; *C. dekopon* × *C. nishinokaori*, Country of Origin: Jeju-island, Republic of Korea) peel (RP), hallabong (*C. reticulata* cv. Shiranui; *C. kiyomi* × *C. ponkan*, Country of Origin: Jeju-island, Republic of Korea) peel (HP), and kumquat (*C. japonica*, Country of Origin: Jeju-island, Republic of Korea) peel (KP). Fruits were purchased from a local grocery market (Jeonju, Republic of Korea). Typical harvest seasons in Korea are as follows: Orange (November–January), Lemon (November–March), Mandarin orange (November–February), Cheonhyehyang (December–March), Hallabong (December–April), Kumquat (February–May), and Redhyang (December–April). Fruits were selected at commercial maturity (fully ripe stage) based on uniform appearance ([e.g., peel colour], size, and absence of visible defects) and were stored at 4 °C until processing. All samples were processed within three days after purchase. CFPs were operationally defined as [flavedo + albedo]. To minimize representativeness bias due to peel thickness and heterogeneous metabolite distribution, peel sampling was standardized by collecting from the equatorial region prior to homogenization. For each cultivar, biological replicates were prepared from independent fruits (*n* = 3 fruits per cultivar). Peels were rinsed with distilled water for 30 s, surface-dried for 30 min, cut into 5 × 5 cm pieces, and homogenized using a pulse blender (BL4258KR, Domestic Electrical Appliances Co., Ltd., China) for 30 s. The homogenate was divided and immediately used for analysis.

### 2.2 OA and FS analyses

The profiling of commercially available CFPs in OA was performed using liquid chromatography–tandem mass spectrometry (LC–MS/MS). Mass spectrometric detection was performed using a Triple Quad™ 4500 LC–MS/MS system (SCIEX, Framingham, MA, USA) with an electrospray ionization source. Samples were prepared by separating peel from CFs, then extracting 1 g of peel with 10 mL of 10% MeOH (Thermo Fisher Scientific Inc., Waltham, MA, USA) containing 500 μL of the internal-standard mixture. The extraction mixture was subjected to a low-frequency sonication system (CPX5800H-E, Emerson Electric Co., St. Louis, MO, USA) for 15 min, followed by vortex mixing for 15 min, and then centrifuged at 14,000 rpm for 10 min at 4 °C using a 5425R instrument (Eppendorf SE, Hamburg, Germany). After centrifugation, 100 μL of the clear supernatant was combined with 100 μL of distilled water (Thermo Fisher Scientific Inc.), and this solution was injected into the LC–MS/MS system. For each sample, 500 μL of citric acid‑*d*_4_ (Alfa Chemistry, Holbrook, NY, USA) (20 ppm) was added as an internal standard for analysis. Seven OAs (quinic acid, aspartic acid, succinic acid, fumaric acid, tartaric acid, malic acid, and citric acid) were employed as internal standards (each at 20 ppm) to ensure quantification accuracy. Chromatographic separation was conducted on an Acclaim™ C30 column (3 μm, 2.1 × 150 mm) maintained at 25 °C. The mobile phase comprised (A) 0.1% formic acid in water and (B) 0.1% formic acid in acetonitrile, delivered at 0.2 mL/min (Meadows et al., 2023). This analysis was conducted six times.

The profiling of commercially available CFPs in FS was performed using a QP-2010 GC–MS (Shimadzu Co., Kyoto, Japan). Peels were separated from CFs, ground, and 3 g of each sample was extracted with 80% MeOH (MS1922–801, Tedia, Fairfield, OH, USA) (2 mL per gram) containing 10 μL of d-sorbitol-13C_6_ internal standard (100 ppm). After centrifugation at 13,000 rpm for 10 min using a VS-180CFI instrument (Vision Scientific Co., Daejeon, Republic of Korea), 100 μL of supernatant was frozen at −110 °C and lyophilized (Tian et al., 2015). The dried residue was derivatized by adding 200 μL pyridine (60200–0380, Junsei Chemical Co., Ltd., Tokyo, Japan) and 100 μL BSTFA (*N*, *O*-bis(trimethylsilyl)trifluoroacetamide) (T6381-10AMP, Sigma-Aldrich, St. Louis, MO, USA), followed by incubating at 56 °C for 30 min in a heating block (VS-251D3, Vision Scientific Co.). Subsequently, 700 μL CHCl_3_ (650471-1 L, Sigma-Aldrich) was added, and the volume was adjusted to 1 mL. GC–MS analysis was conducted on a GC–MS system with an Rxi-5MS column (30 m × 0.25 mm; 0.25 μm) using helium at 1.0 mL/min in split mode (30,1) with a 300 °C injector. The oven temperature was maintained at 70 °C for 3 min, increased at a rate of 10 °C/min to 320 °C, and then held for 7 min, resulting in a total run time of 35 min (Kiweler et al., 2022). Electron ionization was performed at 70 eV, with the ion source at 200 °C and the interface at 230 °C. Data were acquired in SIM mode from 6.00 to 35.00 min, with a solvent cut time of 5.90 min. This analysis was conducted in duplicate.

To determine the contribution of each metabolite to the actual taste detected by LC–MS/MS and GC–MS, taste-active value (TAV) was calculated from taste thresholds and taste contribution (TC) was deducted from TAVs (Liu et al., 2019).

The TAV and TC were calculated using Eqs. (1) and (2) (Liu et al., 2019):

Taste-active value (TAV) = *concentration of OA (or FS)/taste threshold of OA (or FS)* (1).

Taste contribution (TC, %) = *TAV of each flavor metabolite/total TAV* (2).

TAV was calculated as the ratio of the compound concentration to its corresponding taste threshold. TC represents the percentage contribution of each TAV to the total (Liu et al., 2019).

### 2.3 Quantification and calculation of OAs and FSs

Calibration curves for each target OA and FS were established using authentic standards, and analyte concentrations in the analyzed solutions were calculated from the corresponding regression equations. To improve readability, the detailed calibration plots and equations were presented in Fig. S1 and S2.

For FSs, the peak areas of each analyte were normalized to the internal standard, and the concentration in the analyzed solution was determined from the corresponding calibration equation. The calculated value was then corrected for the dilution factor used during sample preparation, converted to the original extract basis using the extraction ratio (2 mL solvent per 1 g sample), and adjusted for the measured solid content during the calculation. The results were expressed as mg/g.

For OAs, the analyte peak area was normalized to the internal standard peak area, and the concentration in the analyzed solution was calculated from the calibration equation. The final content in the peel sample was then obtained by applying the extraction volume (10 mL) and the actual sample mass used for extraction, and the results were expressed as mg/kg.

For each analyte, the concentration in the analyzed solution (C) was calculated using the corresponding calibration equation. The content in the original sample was calculated using the extraction volume (V, mL), the overall dilution factor (DF), and the sample mass (m, g).

For free sugars, the content was calculated as follows:

$$\mathrm{FSs}\;\left(\mathrm{mg}/g\right)=C\times V\times \mathrm{DF}/m$$

where DF includes the dilution applied prior to analysis.

For organic acids, the content was calculated as follows:

$$\mathrm{OAs}\;\left(\mathrm{mg}/\mathrm{kg}\right)=\left(C\times V/m\right)\times 1000$$

where the factor 1000 converts g to kg.

### 2.4 GC–olfactometry–MS analysis

A smart solid-phase micro-extraction fiber (SPME) (DVB/Carbon-wide-range(CWR)/PDMS, 80 μm phase thickness, Agilent Technologies, Santa Clara, CA, USA) mounted on a PAL autosampler was used to collect odor compounds in CFPs. Odor compounds were measured using GC–MS (Agilent 8890 A and 5975C, Agilent Technologies, Santa Clara, CA, USA), and the column of was an HP-5 ms (30 m × 0.25 mm ID × 0.25 μm film thickness). For the measurement, sample (3 g) was placed in a headspace vial and sealed with an aluminum cap. Odor compounds were equilibrated (60 °C, 5 min) in the headspace. Subsequently, the absorption time of odor compounds was for 30 min using a 50/30 μm DVB/C-WR/PDMS gray-coated headspace solid-phase microextracts fiber (HS-SPME, Supelco Inc., Bellefonte, PA, USA) mounted on a PAL auto-sampler. This analysis was conducted in duplicate, and it was possible to reduce the errors during the pretreatment process. The injector temperature was set to 220 °C, and the oven temperature was initially maintained at 40 °C for 5 min, followed by an increase to 200 °C at a rate of 5 °C/min. Helium was used as a carrier gas at a 1.0 mL/min flow rate while operating in the splitless mode. Each separated component in the total ionization chromatogram was identified using a NIST 12 mass spectral library integrated into the mass spectrum. The RI was calculated for qualitative analysis using the *n*-alkane retention time. Pentadecane (C_15:0_, Sigma-Aldrich) was used as the internal standard, and the content (μg/kg) of each odor compound was calculated based on its peak area relative to that of the internal standard (0.005 μg). RI was determined according to Equation (Jo et al., 2025) (1).

(1) $$\mathrm{RI}x=100n+100-\left(\left(\mathrm{tRx}-\mathrm{tRn}\right)/\left(\mathrm{tRn}+1-\mathrm{tRn}\right)\right)$$

where RI*x* denotes the RI of the unknown compound; *tRx* corresponds to the retention time of the unknown compound; *tRn* represents the retention time of the *n*-alkane; and *tRn* + 1 indicates the retention time of the next *n*-alkane. *tRx* lies between *tRn* and *tRn* + 1, where *n* is the number of carbon atoms (Jo et al., 2025).

Each odor-active compound (OAC) was analyzed using a GC–olfactometry system (GC–O; ODP-III; Gerstel, Linthicum, MD, USA). GC–O was conducted simultaneously using GC–MS for 5–25 min, excluding the initial 5 min solvent extraction period. A well-trained sniffer conducted a sniffing test for OACs. Sniffer was initially familiarized with CF varieties and studied the flavor profiling of a typical citrus. The sniffer was pretrained with the odor of the samples and had previous experience with sniffing tests. In addition, the experiment was conducted in an odorless condition, with no olfactory or health issues. The odor intensity of each odor compound was rated on a relative scale of 0–3, with high values indicating strong odor perception (Jo et al., 2025; Lee et al., 2019). During GC–MS analysis, the sniffer was instructed to identify each odor and press a button upon detection. These results were averaged for each flavor compound. OACs were defined as odor compounds that produced a perceivable odor event at the sniffing port, with a detection frequency ≥ 50% across replicate runs, and that were assignable to a GC–MS peak via retention time alignment. Odor descriptors were recorded using a predefined lexicon (e.g., citrus, floral, green/grassy, woody, minty, sweet) after panel training with reference odorants. Odor intensity was scored on a 0–3 scale as described, and only reproducible odor events were retained for downstream OAV/OC calculations. To determine the contribution of each compound to the actual aroma detected by GC–O–MS, odor-active value (OAV) was calculated from odor thresholds and odor contribution (OC) was deducted from OAVs (Jo et al., 2025).

The OAV and OC were calculated using Eqs. (2) and (3) (Jo et al., 2025):

(2) $$\text{Odor}-\text{active value}\;\left(\mathrm{OAV}\right)=\mathrm{concentration of}\;\mathrm{OAC}/\mathrm{odor threshold of}\;\mathrm{OAC}$$

(3) $$\text{Odor contribution}\;\left(\mathrm{OC},\%\right)=\mathrm{OAV}\;\mathrm{of each odor compound}/\mathrm{total}\;\mathrm{OAV}$$

OAV was calculated as the ratio of the compound concentration to its corresponding odor threshold. OC represents the percentage contribution of each OAV to the total (Jo et al., 2025; Liu et al., 2019).

### 2.5 *E*-tongue analysis

An E-tongue (ASTREE II, Alpha MOS, Toulouse, France) was used to analyze the taste profiles of CFPs. The device is equipped with seven sensors: five sensors that correspond to the basic human-perceived tastes, including sourness, saltiness (CTS), umami (NMS), sweetness (ANS), and bitterness (SCS). Five sensors express the relative taste attributes among samples. The sensing system converts electrical signals into digital signals by utilizing the high selectivity of the sensors for specific chemicals and the potential difference between the sensors and a standard electrode (Wang, Wang, et al., 2024).

The CFPs were pulverized using a blender (BL4258KR, Tefal S.A.S., Rumilly, France) prior to use in the experiments. To measure taste attributes, blended peel (5 g) was mixed with purified water (100 mL). The solution was stirred for half-hour (60 °C, 300 rpm) and filtered. Additionally, Solution (10 mL) blended with purified water until 100 mL volume. The experiment time of each sample was 120 s, and cleaning time was 30 s. During the cleaning process, purified water was utilized between sample solutions (Yoon et al., 2024).

### 2.6 *E*-nose analysis

The odor compounds present in the CFPs were analyzed using an *E*-nose (HERACLES Neo, Alpha MOS). Retention time (RT) and index (RI) were identified via AroChemBase (Alpha MOS; containing ≥83,000 compounds) using Kovats indices. The odor compounds were analyzed using the non-polar column (MXT-5). Moreover, the peel (3 g) was placed in a headspace vial (22.5 × 75 mm, PTFE/silicone septum, aluminum cap) and stirred (50 °C, 500 rpm) to equilibrium the odor compounds in the headspace. Odor compounds (5000 μL) were collected through an auto-sampler and analyzed through a flame ionization detector. Incubation temperature, trap absorption temperature, acquisition time, and the flow rate of H^+^ were 50 °C, 40 °C, 227 s, and 1 mL/min, respectively. The oven temperature was increased at 1 °C/s from 50 °C, at 3 °C/s from 80 °C, and held at 250 °C for 21 s (Yoon et al., 2024).

In the present study, the E-nose was primarily used as a comparative aroma-pattern profiling tool to discriminate odor compounds in citrus peel samples. Because pure reference compounds were not used to directly confirm the library-based annotations, these assignments were considered putative identifications and interpreted only at the level of comparative odor pattern analysis. Therefore, the role of the E-nose in this study was to provide rapid fingerprinting of cultivar-dependent odor patterns, whereas GC–MS and GC–O–MS were used to support compound-level identification and interpretation of OACs.

### 2.7 Data processing

The flavor metabolite data are presented as means ± standard deviations. Statistical significance was assessed using SAS version 9.2 (SAS Institute Inc., Cary, NC, USA), with *p* values <0.05 considered significant. A one-way analysis of variance was conducted, followed by pairwise comparisons between samples using Tukey's multiple range test. Principal component analysis (PCA) and hierarchical cluster analysis (HCA) were performed using XLSTAT software version 2024 (Addinsoft, New York, NY, USA) to classify samples based on their flavor compound profiles (Mohamed et al., 2022). PCA produced orthogonal variable sets (principal components, PCs), retaining components with *eigenvalues* > 1.0 according to Kaiser's rule. HCA illustrated the relative dissimilarities among samples in a dendrogram, employing Euclidean distance and Ward's linkage as the agglomeration method (Jo et al., 2025). In addition, to visualize the multivariate analysis results of flavor metabolites, partial least squares discriminant analysis (PLS-DA), pattern hunter analysis, and the Debiased Sparse Partial Correlation (DSPC) network were performed using the MetaboAnalyst 6.0 software (https://www.metaboanalyst.ca).

## 3 Results and discussion

### 3.1 Taste compounds analysis

This study compared and analyzed the taste patterns of seven different CFPs using an *E*-tongue. In addition, the contents of FSs and OAs—key taste compounds associated with AHS_sourness and ANS_sweetness responses—as well as the characteristic taste attributes of CFPs among the five basic tastes, were compared and analyzed. Citric acid and malic acid are the major OAs in CFPs, while fructose, glucose, and sucrose are known to be the major FSs in CFPs (Guo et al., 2018; Jo et al., 2025).

#### 3.1.1 FS analysis

**Fig. 1:**
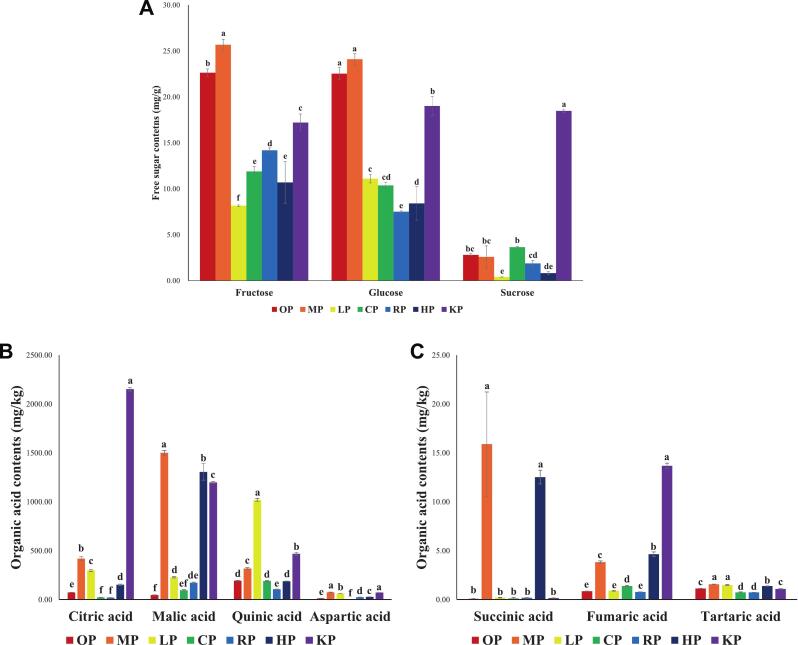
(A) FS and (B and C) OA contents of citrus fruit peels (CFPs), respectively. OP: orange peel, MP: mandarin orange peel, LP: lemon peel, CP: cheonhyehyang peel, RP: redhyang peel, HP: hallabong peel, and KP: kumquat peel. (For interpretation of the references to colour in this figure legend, the reader is referred to the web version of this article.)

Generally, fructose, glucose, and sucrose are known as the top three FSs with high ANS (Wee et al., 2018). The results of the FS content analyzed in this study are presented in Table S1 and Fig. 1A. Fructose was detected at the highest concentration in MP (25.68 ± 0.60 mg/g), followed by OP and KP (*p* < 0.05). By contrast, the lowest fructose concentration was observed in LP (8.16 ± 0.09 mg/g; *p* < 0.05). Similarly, glucose showed the highest concentration in MP (24.11 ± 0.62 mg/g), followed by OP and KP (*p* < 0.05), while the lowest glucose concentration was found in RP (7.51 ± 0.08 mg/g, *p* < 0.05). Additionally, sucrose was detected at the highest concentration of 18.48 ± 0.19 mg/g in KP and the lowest concentration of 0.40 ± 0.02 mg/g in LP (*p* < 0.05).

#### 3.1.2 OA analysis

The results of the OA content analysis are presented in Table S1, Fig. 1B and C. Citric acid was detected at the highest content of 2152.38 ± 18.84 mg/kg in KP and the lowest content of 20.54 ± 0.89 mg/kg in RP (*p* < 0.05). Malic acid was detected at the highest concentration of 1501.55 ± 23.88 mg/kg in MP and the lowest concentration of 46.30 ± 1.02 mg/kg in OP (*p* < 0.05). Quinic acid was detected at the highest concentration of 1018.75 ± 15.90 mg/kg in LP and the lowest concentration of 105.37 ± 2.48 mg/kg in RP (*p* < 0.05). Aspartic acid was detected at the highest concentration of 73.88 ± 2.04 mg/kg in MP and the lowest concentration of 1.62 ± 0.25 mg/kg in CP (*p* < 0.05). Succinic acid was detected at the highest concentration of 12.53 ± 0.71 mg/kg in MP and the lowest concentration of 0.08 ± 0.00 mg/kg in OP (*p* < 0.05). Fumaric acid was detected at the highest concentration of 13.69 ± 0.27 mg/kg in KP and the lowest concentration of 0.78 ± 0.02 mg/kg in RP (*p* < 0.05). Tartaric acid was detected at the highest concentration of 1.56 ± 0.02 mg/kg in MP and the lowest concentration of 0.74 ± 0.01 mg/kg in RP (*p* < 0.05).

#### 3.1.3 Taste pattern analysis using *E*-tongue

**Fig. 2:**
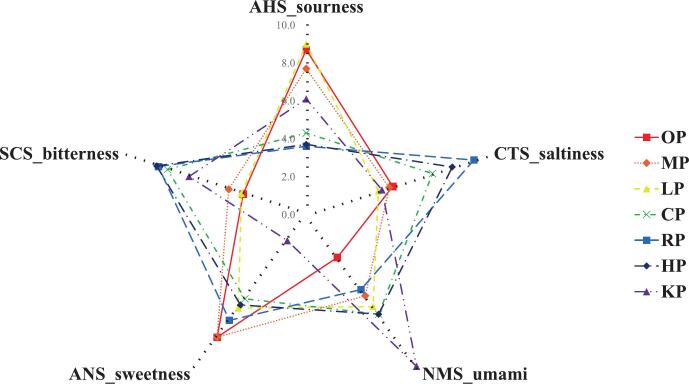
Taste pattern screening of citrus fruit peels (CFPs) analyzed using an E-tongue. OP: orange peel, MP: mandarin orange peel, LP: lemon peel, CP: cheonhyehyang peel, RP: redhyang peel, HP: hallabong peel, and KP: kumquat peel. (For interpretation of the references to colour in this figure legend, the reader is referred to the web version of this article.)

The results of the taste patterns analyzed by the E-tongue are shown in Fig. 2. The sourness intensity was highest in LP (9.0) and lowest in RP (3.6). The CTS intensity was highest in RP (9.3) and lowest in LP (4.0). The NMS intensity was highest in KP (9.9) and lowest in OP (2.8). The ANS intensity was highest in OP (8.0) and MP (8.0) and lowest in KP (1.7). Finally, the SCS intensity was highest in HP (8.3) and lowest in OP (3.5). OP and MP exhibited relatively high concentrations of fructose and glucose in the FS analysis compared to other CFPs (*p* < 0.05), which is believed to contribute to their elevated ANS intensity (8.0). By contrast, although KP showed a relatively high sucrose concentration (*p* < 0.05), its ANS intensity was the lowest (1.7), indicating that sucrose may have a limited influence on ANS activation in CFPs. These findings indicate that fructose and glucose play a key role in influencing ANS responses compared with sucrose. OAs in CF are widely used in the food, beverage, pharmaceutical, and health supplement industries, giving them significant commercial value. For instance, citric acid is commonly added to fruit drinks as an acidulant to improve nutritional content and palatability (Liew et al., 2018). In the OA analysis, LP exhibited the highest quinic acid concentration among the CFPs (*p* < 0.05), which corresponds to its elevated sourness intensity (9.0). In addition, various soluble components can influence the taste pattern of CFP. Among the sensors of the E-tongue, sourness and ANS are attributed to OAs and FSs, respectively (Jo et al., 2025). Notably, RP exhibited the lowest quinic acid concentration among the CFPs (*p* < 0.05), which corresponded to its reduced sourness intensity (3.6). By contrast, although KP had the highest citric acid concentration among the OAs (*p* < 0.05), its impact on sourness intensity was minimal. This finding showed a different trend from previous research on citric acid, which indicated that citric acid primarily affects the sourness of CFs (Albertini et al., 2006). Therefore, major OAs present in CFPs, such as citric, malic, and quinic acid have the most significant influence on the sour taste of CFPs (Albertini et al., 2006).

### 3.2 Odor compound analysis using GC–MS

**Fig. 3:**
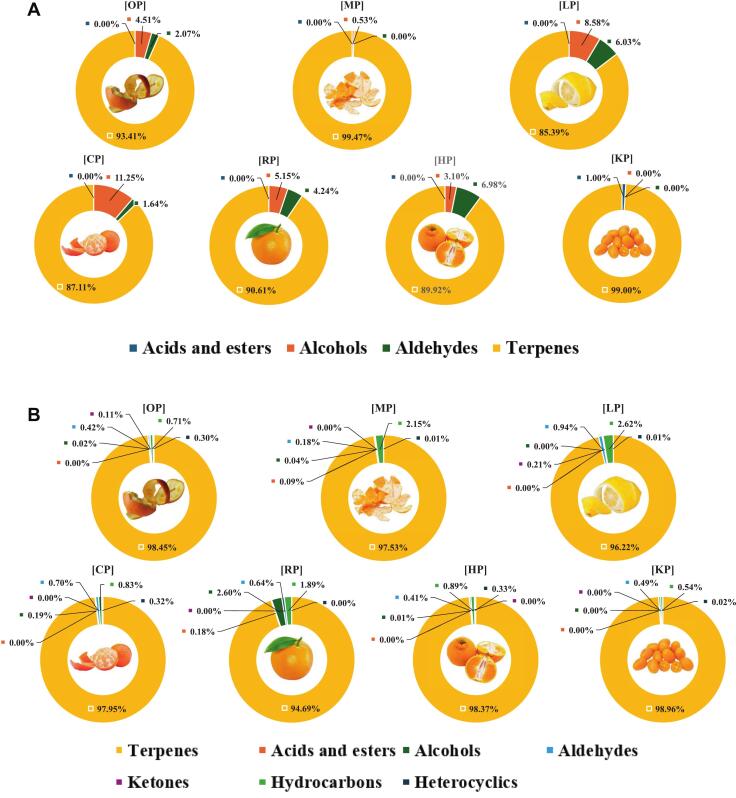
Pie charts of odor compounds in citrus fruit peels (CFPs) analyzed using (A) E-nose and (B) GC–MS. OP: orange peel, MP: mandarin orange peel, LP: lemon peel, CP: cheonhyehyang peel, RP: redhyang peel, HP: hallabong peel, and KP: kumquat peel. (For interpretation of the references to colour in this figure legend, the reader is referred to the web version of this article.)

Odor compounds in seven CFPs were analyzed using GC–MS, and results are shown in Fig. 3A and Table 1. All odor compounds detected by the GC–MS system were classified into the following groups: 2 acids and esters, 12 alcohols, 7 aldehydes, and 32 terpenes. The number of odor compounds in the other CFPs was as follows: OP (17), MP (21), CP (21), RP (18), and HP (20). LP exhibited the highest number (23) of odor compounds, whereas KP exhibited the lowest (13).

**Table 1:** Volatile compounds of citrus fruit peel analyzed by GC–MS (μg/kg).1)

Volatile compounds	RT^1)^(min)	RI^2)^	OP	MP	LP	CP	RP	HP	KP	I.D.^3)^
**Acids and esters (2)**										
n-Octyl acetate	22.40	1225.36	ND^4)^	ND	ND	ND	ND	ND	12.42 ± 2.56	MS
Geranyl acetate	26.97	1398.88	ND	ND	ND	ND	ND	ND	18.71 ± 26.46	MS
										
**Alcohols (12)**										
3-Hexenol	11.63	877.29	ND	19.05 ± 0.74	ND	6.51 ± 0.24	ND	6.41 ± 0.20	ND	MS/RI^5)^
1-Hexanol	12.01	887.58	ND	ND	ND	12.23 ± 0.32	ND	5.36 ± 0.58	ND	MS
3-Methylpentanol	12.02	887.76	ND	38.49 ± 0.19	ND	ND	ND	ND	ND	MS
Linalool	19.41	1120.26	163.63 ± 231.41	ND	176.99 ± 250.30	171.61 ± 54.88	261.12 ± 0.96	ND	ND	MS/RI
Menthadienol	20.44	1156.64	ND	ND	ND	38.56 ± 2.09	ND	ND	ND	MS
Terpinen-4-ol	21.65	1197.09	ND	ND	264.24 ± 2.94	ND	ND	ND	ND	MS/RI
*α*-Terpineol	22.01	1210.49	134.70 ± 9.64	ND	331.64 ± 27.31	39.51 ± 0.51	39.88 ± 1.15	ND	ND	MS/RI
Carveol	22.83	1241.73	ND	ND	ND	139.08 ± 5.92	ND	ND	ND	MS/RI
Citronellol	22.92	1245.01	212.65 ± 6.24	ND	569.26 ± 45.68	17.14 ± 0.76	32.61 ± 1.68	37.24 ± 0.44	ND	MS/RI
Geraniol	23.67	1272.52	94.41 ± 6.38	ND	628.14 ± 46.94	ND	ND	ND	ND	MS/RI
Limonen-10-ol	24.78	1313.65	ND	ND	ND	131.94 ± 2.61	ND	ND	ND	MS
3,7-Dimethyl-2,6-octadienol	26.59	1384.87	ND	ND	244.71 ± 346.07	ND	ND	ND	ND	MS
										
**Aldehydes (7)**										
Hexanal	9.85	823.91	ND	ND	ND	ND	10.68 ± 0.44	ND	ND	MS/RI
2-Hexenal	11.52	874.01	27.32 ± 2.15	ND	ND	ND	10.05 ± 0.04	ND	ND	MS/RI
Citronellal	20.93	1173.27	ND	ND	234.57 ± 2.69	ND	93.41 ± 1.36	25.53 ± 1.56	ND	MS/RI
Decanal	22.32	1222.12	131.84 ± 1.61	ND	88.74 ± 2.33	69.51 ± 0.61	139.09 ± 3.39	67.42 ± 1.26	ND	MS
Neral	23.35	1260.72	27.69 ± 39.16	ND	465.95 ± 9.62	ND	ND	ND	ND	MS/RI
Citral	24.13	1288.69	91.45 ± 8.55	ND	770.24 ± 81.10	ND	ND	ND	ND	MS/RI
Dodecanal	27.62	1426.56	ND	ND	ND	11.52 ± 0.84	21.51 ± 1.07	17.30 ± 0.56	ND	MS
										
**Terpenes (32)**										
*α*-Pinene	14.16	953.81	1077.85 ± 12.40	408.58 ± 5.85	1486.16 ± 172.72	303.09 ± 102.40	560.21 ± 20.58	133.11 ± 188.25	37.50 ± 53.03	MS/RI
Camphene	14.77	971.91	ND	ND	427.41 ± 10.31	ND	ND	ND	ND	MS/RI
*β*-Pinene	15.58	994.84	ND	359.89 ± 2.05	ND	ND	200.79 ± 4.75	ND	ND	MS/RI
2,7-Dimethyl-oct-3-en-5-yne	15.72	998.67	ND	ND	1778.42 ± 2515.06	ND	ND	ND	ND	MS
*β*-Myrcene	16.08	1010.68	ND	470.33 ± 2.49	ND	396.73 ± 11.81	638.25 ± 13.14	393.95 ± 59.47	246.38 ± 21.87	MS
*β*-Phellandrene	16.39	1021.60	ND	63.16 ± 2.18	ND	ND	ND	37.66 ± 53.26	18.65 ± 2.50	MS/RI
*α*-Phellandrene	16.57	1027.48	ND	94.10 ± 133.08	ND	ND	ND	ND	26.69 ± 0.07	MS/RI
Limonene	17.43	1055.85	8675.61 ± 7804.34	6208.67 ± 93.90	10,491.04 ± 165.28	3195.19 ± 837.16	3225.75 ± 4227.11	485.01 ± 0.84	2627.90 ± 3384.49	MS/RI
3-Carene	18.33	1084.08	2080.18 ± 462.75	ND	4110.77 ± 458.29	60.37 ± 85.37	ND	ND	ND	MS
*γ*-Terpinene	18.51	1089.49	ND	ND	ND	126.43 ± 2.93	1165.14 ± 3.18	67.95 ± 96.09	ND	MS/RI
2-Carene	19.13	1109.95	340.51 ± 481.55	1909.01 ± 65.95	2323.93 ± 3286.54	100.82 ± 4.31	25.90 ± 1.98	89.57 ± 2.65	28.84 ± 40.78	MS
*δ*-Terpinene	19.37	1118.88	ND	317.24 ± 111.41	390.96 ± 3.73	ND	ND	ND	ND	MS/RI
1,3,8-Menthatriene	19.79	1133.84	ND	ND	ND	27.02 ± 38.22	ND	ND	ND	MS
Limonene oxide	20.54	1159.83	204.61 ± 8.47	95.56 ± 29.10	ND	44.51 ± 1.28	38.76 ± 0.71	36.18 ± 0.08	ND	MS/RI
Carvone	23.53	1267.35	ND	ND	ND	42.29 ± 4.48	ND	14.56 ± 0.78	ND	MS/RI
2,6-Dimethyl 2,6-octadiene	26.19	1369.39	ND	7.53 ± 10.65	82.59 ± 1.69	ND	ND	12.96 ± 0.16	ND	MS
*α*-Cubebene	26.26	1372.23	ND	33.25 ± 1.72	ND	ND	ND	ND	6.39 ± 4.39	MS/RI
Copaene	27.01	1400.61	52.62 ± 0.20	70.85 ± 3.49	ND	7.62 ± 2.69	4.22 ± 0.16	3.44 ± 0.66	ND	MS/RI
Caryophyllene	28.15	1449.10	46.17 ± 0.84	123.45 ± 17.18	430.72 ± 284.10	ND	ND	3.38 ± 0.11	ND	MS/RI
*α*-Bergamotene	28.45	1461.50	ND	ND	109.49 ± 30.56	ND	ND	ND	ND	MS/RI
Aromandendrene	28.63	1468.59	ND	ND	ND	ND	9.92 ± 0.49	4.05 ± 0.04	ND	MS
*δ*-Muurolene	28.79	1475.35	ND	149.36 ± 19.88	ND	ND	ND	ND	ND	MS
*β*-Farnesene	28.80	1475.68	ND	ND	43.92 ± 62.11	ND	ND	ND	ND	MS/RI
*α*-Guaiene	28.87	1478.52	ND	18.85 ± 1.03	ND	ND	ND	ND	ND	MS/RI
*β*-Ocimene	28.95	1481.88	ND	ND	ND	ND	ND	ND	6.32 ± 1.22	MS
Humulene	29.03	1484.90	ND	92.37 ± 4.46	ND	ND	ND	ND	ND	MS/RI
*β*-Bisabolene	29.15	1489.97	ND	4.45 ± 6.30	467.35 ± 80.12	ND	ND	ND	ND	MS/RI
*γ*-Muurolene	29.45	1502.04	ND	ND	ND	ND	1.42 ± 2.01	16.20 ± 1.48	40.68 ± 14.78	MS/RI
Isoledene	29.53	1505.85	ND	ND	ND	ND	ND	ND	5.54 ± 7.83	MS
Germacrene D	29.61	1509.33	18.74 ± 26.50	70.49 ± 2.54	ND	ND	ND	ND	22.30 ± 2.92	MS/RI
*α*-Bisabolene	30.01	1527.12	33.01 ± 0.04	ND	114.69 ± 6.29	ND	ND	ND	ND	MS
*α*-Farnesene	30.07	1529.66	ND	235.19 ± 38.87	ND	6.11 ± 0.27	ND	122.71 ± 2.35	ND	MS/RI

1 RT: retention time, ^2)^RI: retention index, ^3)^I.D.: identification, ^4)^ND: not detected, ^5)^MS/RI: identified compounds using both MS and RI.

Fig. 3A shows the ratio (%) of odor compound groups in each CFP. The composition of terpenes in all CFPs was 85.39%–99.47% among all odor compounds. Previous research indicated that CFP accounted for >90% of the terpene peak area, findings similar to those of this study (Ren et al., 2015). However, the actual impact on the scent perceived by humans is less than the actual concentration due to the high odor threshold (Jo et al., 2025; Ren et al., 2015). Among the terpenes, limonene exhibited the highest concentration and is known for its characteristic citrus-like aroma (Liang et al., 2022). Limonene was detected in all CFPs, and limonene showed the highest concentration (10,491.04 ± 165.28 μg/kg) in LP and the lowest concentration (485.01 ± 0.84 μg/kg) in RP. According to Lopresto et al. (2014), LP has higher limonene concentration than that of other genotypes in the same grove. *α*-Pinene was detected in all CFPs and exhibits citrus, spicy, and woody pine odors (Vespermann et al., 2017). *α*-Pinene showed the highest concentration (1486.16 ± 172.72 μg/kg) in LP and the lowest concentration (37.50 ± 53.03 μg/kg) in KP. Furthermore, *β*-pinene, an isomer of α-pinene that exhibits cool woody, fresh mint, and camphor odors, was detected only in MP and RP (Vespermann et al., 2017). The concentrations of *β*-pinene in MP and RP were identified to be 359.89 ± 2.05 and 200.79 ± 4.75 μg/kg, respectively. Furthermore, 2-carene was detected in all CFPs and is recognized as a major odor compound of pine essential oil (Woo et al., 2019). The highest concentration (2323.93 ± 3286.54 μg/kg) was found in LP, whereas the lowest concentration (25.90 ± 1.98 μg/kg) was found in RP. In addition, 3-carene (bicyclic monoterpene), an isomer of 2-carene, has a sweet and spicy odor and was detected only in OP, LP, and CP (Woo et al., 2019). The highest concentration (4110.77 ± 458 μg/kg) was detected in LP, and the lowest concentration (60.37 ± 85.37 μg/kg) was detected in CP. Weng et al. (2024) reported that *γ*-terpinene was the second most abundant odor compound in the essential oil of *C. depressa* peel and noted its association with a citrus-like aroma. *γ*-Terpinene was detected at the highest concentration (1165.14 ± 3.18 μg/kg) in RP and at the lowest concentration (67.95 ± 96.09 μg/kg) in HP. Among the odor compounds detected in most CFPs, *β*-myrcene was identified, and this monoterpene, which possesses a pleasant odor, is used as a flavor enhancer in the perfume manufacturing, alcoholic beverage, and food industries (Bonamin et al., 2014). The highest concentration (638.25 ± 13.14 μg/kg) was detected in RP, whereas the lowest concentration (246.38 ± 21.87 μg/kg) was detected in KP.

Consequently, the abundant odor compound group was identified as terpenes, and limonene, *α*-pinene, *β*-pinene, 2-carene, 3-carene, *γ*-terpinene, and *β*-myrcene were identified as major odor compounds in CFPs. Previous studies have shown that odor compound profiles of CF using GC–MS are similar to our results (Jo et al., 2025; Ren et al., 2015; Zhang et al., 2017).

### 3.3 Odor compound analysis using *E*-nose

Fig. 3B shows the odor compound profiles of seven CFPs analyzed using an E-nose, while detailed peak areas are represented in Table 2. A total of 60 odor compounds were identified, comprising 7 terpenes, 6 acids and esters, 13 alcohols, 6 aldehydes, 4 ketones, 9 hydrocarbons, 8 heterocyclic compounds, and 7 others. In addition, 13 odor compounds of OP, 14 odor compounds of MP, 16 odor compounds of LP, 21 odor compounds of CP, 15 odor compounds of RP, 17 odor compounds of HP, and 10 odor compounds of KP were detected.

**Table 2:** Volatile compounds of citrus fruit peels analyzed by E-nose (peak area × 10^3^).1)

Compounds	RT^1)^(RI^2)^)	Sensory description	OP	MP	LP	CP	RP	HP	KP
**Terpenes (7)**									
*α*-Pinene	56.79(938)	Fresh, Pine	84.99 ± 3.24	57.85 ± 2.50	109.66 ± 1.69	ND^3)^	ND	ND	ND
*β*-Pinene	61.31(988)	Aromatic	ND	ND	ND	449.11 ± 188.09	ND	789.74 ± 7.56	ND
Myrcene	61.99(996)	Fruity, Lemon	267.49 ± 7.66	288.32 ± 11.87	1480.66 ± 31.50	3.48 ± 4.92	ND	8.62 ± 0.07	199.96 ± 14.85
Limonene	65.34(1047)	Citrus, Orange	10,242.14 ± 177.09	8909.56 ± 297.11	8966.42 ± 341.85	10,580.33 ± 245.33	10,855.46 ± 9.14	10,688.07 ± 134.51	10,049.40 ± 515.72
Terpinolene	67.51(1075)	Citrus, Fruity	ND	ND	ND	45.43 ± 12.64	316.74 ± 0.90	78.87 ± 2.02	24.30 ± 2.10
Linalool	69.58(1105)	Floral, Citrus	ND	ND	ND	ND	ND	16.11 ± 2.57	ND
*β*-Ionone	88.53(1463)	Floral, Powdery	ND	ND	ND	ND	33.78 ± 12.20	37.92 ± 17.71	ND
									
**Acids and esters (6)**									
Vinyl acetate	21.74(586)	Sharp	ND	0.03 ± 0.04	ND	ND	ND	ND	ND
Propyl acetate	33.17(715)	Fruity	ND	ND	ND	ND	0.07 ± 0.02	ND	ND
Methyl crotonate	38.89(763)	Fruity, Green	ND	ND	ND	0.12 ± 0.09	ND	ND	ND
Ethyl butyrate	42.83(796)	Fruity, Sweet	ND	ND	ND	0.05 ± 0.06	ND	ND	ND
Ethyl 2-methylbutyrate	48.42(851)	Apple, Green	ND	8.05 ± 0.35	ND	ND	ND	ND	ND
Hexanoic acid	62.77(1007)	Sour	ND	ND	ND	ND	21.40 ± 0.10	ND	ND
									
**Alcohols (13)**									
2-Methyl-2-propanol	17.42(489)	Camphor	0.97 ± 0.21	ND	ND	ND	ND	ND	ND
2-Methylpropanol	18.12(505)	Fresh, Green	0.96 ± 0.80	ND	ND	ND	ND	ND	ND
1-Propanol	20.02(547)	Fruity	ND	ND	0.11 ± 0.15	ND	ND	ND	ND
2-Butanol	21.88(588)	Sulfurous	ND	ND	ND	ND	0.42 ± 0.33	0.11 ± 0.04	ND
Butanol	27.06(652)	Fruity	ND	ND	0.03 ± 0.04	ND	ND	ND	ND
Trometamol	29.08(675)	Sweet, Pungent	ND	ND	0.03 ± 0.04	ND	ND	ND	ND
Pent-1-en-3-ol	29.68(680)	Fruity, Grassy	ND	ND	ND	0.44 ± 0.00	0.30 ± 0.08	0.16 ± 0.03	ND
3-Pentanol	31.34(700)	Fruity, Green	0.31 ± 0.04	ND	ND	ND	ND	0.40 ± 0.06	ND
3-Methyl-1-butanol	35.31(733)	Fruity	ND	ND	ND	0.06 ± 0.01	ND	ND	ND
2-Methyl-1-butanol	35.84(737)	Fresh, Fruity	ND	ND	ND	ND	ND	ND	0.08 ± 0.03
Cyclohexanol	51.73(880)	Camphor	ND	ND	ND	20.96 ± 8.60	ND	ND	ND
Heptan-2-ol	53.58(900)	Fresh, Green	ND	4.08 ± 0.10	ND	ND	ND	ND	ND
3-Octanol	61.83(994)	Citrus	ND	ND	ND	ND	307.53 ± 1.46	ND	ND
									
**Aldehydes (6)**									
Acetaldehyde	15.07(436)	Fresh, Fruity	44.77 ± 11.50	ND	37.92 ± 6.72	30.01 ± 15.93	41.80 ± 7.58	32.95 ± 4.46	7.29 ± 0.76
Propenal	16.33(465)	Sweet	ND	17.14 ± 3.58	ND	46.64 ± 12.32	8.71 ± 0.69	11.33 ± 1.81	43.09 ± 0.29
Butanal	20.82(565)	Green, Musty	ND	0.11 ± 0.15	0.05 ± 0.07	ND	ND	ND	ND
Hexanal	41.29(785)	Fruity	0.10 ± 0.14	ND	ND	2.59 ± 0.41	24.90 ± 2.17	4.01 ± 0.45	ND
3-Hexanal	41.75(787)	Apple, Fruity	ND	ND	ND	0.10 ± 0.14	ND	ND	ND
Benzeneacetaldehyde	63.54(1021)	Floral, Grassy	ND	ND	64.91 ± 1.60	ND	ND	ND	ND
									
**Ketones (4)**									
1-Hydroxy-2-propanone	26.68(647)	Sweet, Pungent	ND	ND	ND	ND	ND	0.04 ± 0.06	ND
Acetoin	31.81(703)	Butter, Dairy	ND	ND	ND	0.40 ± 0.03	ND	ND	ND
3-Hexen-2-one	47.66(844)	–	11.61 ± 0.45	ND	ND	ND	ND	ND	ND
*δ*-Valerolactone	59.40(969)	–	ND	ND	23.26 ± 0.32	ND	ND	ND	ND
									
**Hydrocarbons (9)**									
2-Methylbutane	15.16(439)	Pleasant	ND	28.22 ± 5.44	16.00 ± 2.89	ND	ND	ND	ND
Heptane	31.40(701)	Fruity	ND	0.88 ± 0.12	ND	ND	ND	ND	4.61 ± 0.75
1-Chloropentane	38.43(759)	Sweet	ND	ND	ND	ND	ND	ND	0.24 ± 0.01
3-Methyl-octane	52.43(887)	–	ND	ND	ND	ND	52.94 ± 1.35	ND	ND
1,1,1,2-Tetrachloroethane	54.00(905)	–	ND	ND	7.91 ± 0.24	ND	ND	ND	ND
4-Methylnonane	57.72(949)	–	ND	140.89 ± 5.98	263.63 ± 1.96	94.34 ± 2.85	170.90 ± 1.03	104.61 ± 1.59	51.30 ± 4.71
Glycerol	59.18(966)	Bitter	ND	5.46 ± 0.36	ND	2.63 ± 0.08	ND	ND	ND
5-Methyl-4-nonene	62.42(1020)	–	ND	28.25 ± 0.55	ND	ND	ND	ND	ND
Pentylcyclopentane	64.10(1029)	–	76.04 ± 0.21	ND	ND	ND	ND	ND	ND
									
**Heterocyclics (8)**									
Furan	17.96(501)	Etheral	ND	1.17 ± 0.20	ND	ND	ND	ND	ND
2-Methylfuran	22.48(600)	Grassy	0.40 ± 0.01	ND	ND	ND	ND	0.26 ± 0.00	ND
Pyrrole	37.38(750)	Nutty, Sweet	ND	ND	ND	ND	ND	0.11 ± 0.03	ND
2-Methylthiophene	40.26(776)	Green	0.40 ± 0.10	ND	0.57 ± 0.26	ND	ND	ND	ND
m-Xylene	51.54(878)	Aromatic	ND	ND	ND	ND	ND	39.12 ± 0.79	ND
Phenol	59.77(970)	Aromatic, Sweet	ND	ND	ND	ND	ND	ND	1.67 ± 0.76
4-Octanolide	78.02(1253)	Coconut, Dairy	31.64 ± 1.32	ND	ND	ND	ND	ND	ND
Geosmin	88.65(1466)	Earthy, Musty	ND	ND	ND	35.72 ± 16.98	ND	ND	ND
									
**Other compounds (7)**									
Diethyl ether	17.53(492)	Etheral	ND	ND	0.79 ± 0.14	1.24 ± 0.24	1.24 ± 0.06	ND	ND
2-Methyl-2-propanethiol	21.94(589)	Sulfurous	ND	ND	ND	0.11 ± 0.05	ND	ND	ND
Ethane, 1,2-dichloro	22.65(602)	Sweet	ND	ND	ND	0.33 ± 0.10	ND	ND	ND
1,2-Dichloropropane	31.60(703)	Sweet	ND	ND	0.39 ± 0.05	ND	ND	ND	ND
Glycerol	59.55(968)	Almond, Bitter	ND	ND	ND	2.63 ± 0.08	ND	ND	ND
Decalin	69.47(1103)	–	ND	ND	ND	ND	32.40 ± 1.38	ND	ND
1-Octanethiol	71.47(1136)	Sulfurous	ND	ND	ND	15.01 ± 3.44	ND	ND	ND

1 RT: retention time, ^2)^RI: retention index, ^3)^ND: not detected.

Among all CFPs, the highest peak area of limonene was observed in RP (10,855.46 ± 9.14), and the lowest peak area was detected in MP (8909.56 ± 297.11). Furthermore, *α*-pinene was detected only in OP (84.99 ± 3.24), MP (57.85 ± 2.50), and LP (109.66 ± 1.69), while *β*-pinene was detected only in CP (449.11 ± 188.09) and HP (789.74 ± 7.56). In addition, myrcene was detected in all CFPs, except for RP. Among all CFPs, the highest peak area was detected in LP (1480.66 ± 31.50), and the lowest peak area was detected in CP (3.48 ± 4.92). In addition, terpinolene is a structural isomer of limonene, a monocyclic monoterpene containing a cyclohexane ring and having a pine odor. Terpinolene was detected in all CFPs except OP, MP, and LP, with the highest peak area in RP (316.74 ± 0.90) and the lowest in KP (24.30 ± 2.10). This tendency may be attributed to the isomeric relationship (positive correlation) between limonene and terpinolene (Sowndhararajan et al., 2015). As previously mentioned, the total peak areas of terpenes were the highest in RP and the lowest in KP. Among the odor compounds, limonene, myrcene, terpinolene, and *β*-pinene contributed most significantly to the overall peak area.

Therefore, these odor compounds may be considered characteristic markers detected by the *E*-nose in the CFPs analyzed in this study. The difference between the results of the odor compounds analyzed using the E-nose and GC–MS was that more terpene odor compounds were detected through GC–MS, and the ratio of key odor compounds within the CFP differed. These differences are attributed to fundamental analytical principles and to variations in sensitivity between the two techniques. In the E-nose analysis method, the Kovats retention index library enables the comparison of odor patterns and rapid profiling of odor compounds based on retention index information. However, the library has inherent limitations in accurately identifying and quantifying individual odor compounds, particularly because pure reference compounds were not used to directly confirm the library-based annotations in the present study. Therefore, these assignments should be regarded as putative identifications. By contrast, GC–MS separates the complex odor compounds in the sample over time and then identifies their molecular weight and structure. This provides qualitative and quantitative analysis of individual odor compounds, offering high accuracy, reproducibility, and reliability (Moon et al., 2025). Due to these analytical differences, the E-nose offers advantages, including rapid profiling and pattern-based comparisons, whereas GC–MS is better suited for precise identification and quantification of complex odor compound mixtures. Therefore, the integrated application of these two techniques can provide a more multifaceted and comprehensive understanding of the aroma characteristics of CFPs. Complementary approaches may further clarify the specific features of the odor compounds present in CFPs (Zhang et al., 2024).

### 3.4 OACs in CFPs

**Fig. 4:**
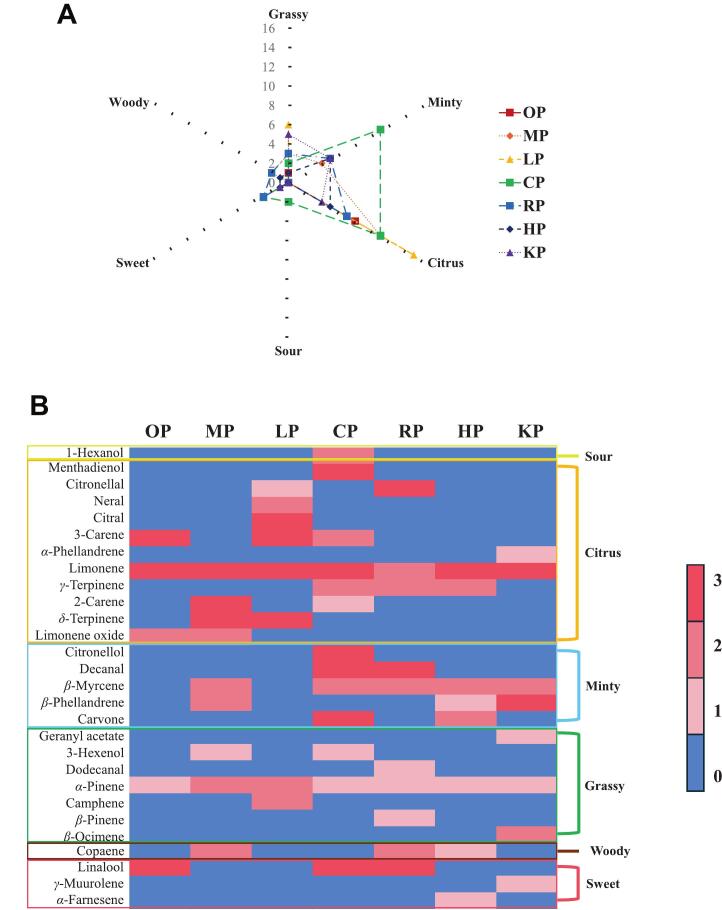
(A) Over GC-O results and (B) odor intensities of odor-active compounds (OACs) analyzed using GC–O. OP: orange peel, MP: mandarin orange peel, LP: lemon peel, CP: cheonhyehyang peel, RP: redhyang peel, HP: hallabong peel, and KP: kumquat peel. (For interpretation of the references to colour in this figure legend, the reader is referred to the web version of this article.)

OACs of CFPs were analyzed through the GC–O system. GC-O results are shown in Fig. 4A, and results of odor intensities are shown in Fig. 4B. A total of 28 OACs were identified in this study, and in cases of different OACs sharing similar or identical odors, the odor intensities were combined to calculate the total odor intensity (Fig. 4A). The OACs associated with grassy odors were geranyl acetate, 3-hexenol, dodecanal, *α*-pinene, camphene, *β*-pinene, and *β*-ocimene. LP exhibited the highest grassy odor intensity (6), whereas OP and HP showed the lowest intensity (1). OACs associated with mint were identified as citronellol, decanal, *β*-myrcene, *β*-phellandrene, and carvone. CP exhibited the highest minty odor intensity (11), followed by RP, HP, and KP (each with an intensity of 5), and MP (intensity of 4). The remaining CFPs showed no detectable minty aroma. The highest citrus odor intensity was perceived as 15 in LP, and the lowest was perceived as 4 in KP. The OAC perceived as a sour odor was 1-hexanol, and this compound was the only one perceived as intensity 2 in CP. The OACs perceived as sweet were identified as linalool, *γ*-muurolene, and *α*-farnesene. Sweet odors exhibited the highest intensity (3) in OP, CP, and RP, and a lower intensity (1) in HP and KP. Sweet odor was not detected in MP and LP. In addition, the OAC associated with woody aroma was identified as copaene, which was only recognized as having an intensity of 2 in MP and RP. Consequently, the OACs associated with the citrus odor, which is the major flavor and distinguishes between CFs and other fruits, were identified as menthadienol, citronellal, neral, citral, 3-carene, *α*-phellandrene, limonene, *γ*-terpinene, 2-carene, *δ*-terpinene, and limonene oxide (Sharon-Asa et al., 2003).

### 3.5 Flavor contribution and flavor codes in CFPs

**Fig. 5:**
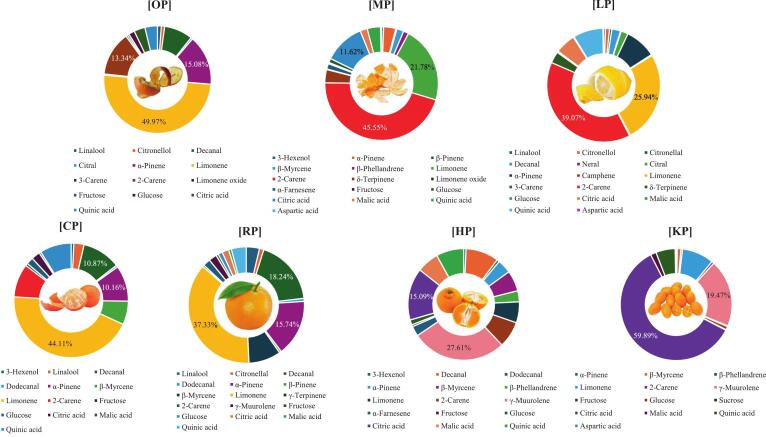
Flavor codes in citrus fruit peels (CFPs) to taste and odor profiles. OP: orange peel, MP: mandarin orange peel, LP: lemon peel, CP: cheonhyehyang peel, RP: redhyang peel, HP: hallabong peel, and KP: kumquat peel. (For interpretation of the references to colour in this figure legend, the reader is referred to the web version of this article.)

In the case of flavor code of each CFPs, OAV and TAV were calculated to identify the flavor contribution of each flavor metabolite to the CFPs. These were calculated based on the thresholds of taste or odor using the concentrations of OACs, FSs, and OAs analyzed by chromatographic approaches. Furthermore, flavor metabolites with an active value of ≥1 are considered as key contributors to the overall aroma profile (Yoon et al., 2024). Contribution values (OC and TC) represent the contribution to total flavor (percentage). Flavor metabolites with a contribution of 10% or higher were significant considered (Jo et al., 2025; Liu et al., 2019). The active values (OAV and TAV), contribution values (OC and TC), and thresholds of flavor metabolites are presented in Table S2. In addition, the contribution of flavor metabolites (OC and TC) affecting the flavor of each CFP is shown in Fig. 5. The flavor of OP comprised 90.53% OC and 9.47% TC. Among these, the significant metabolites were limonene (49.97%), α-pinene (15.08%), and 2-carene (13.34%). The flavor of MP comprised 80.69% OC and 19.31% TC. Among these, the significant metabolites were 2-carene (45.55%), limonene (21.78%), and citric acid (13.62%). The flavor of LP comprised 84.82% OC and 15.18% TC. Among these, the significant metabolites were 2-carene (39.07%) and limonene (25.94%). The flavor of CP comprised 85.43% OC and 14.57% TC. Among these, limonene (44.11%), decanal (10.87%), and *α*-pinene (10.16%) were significant metabolites. The flavor of RP was indicated to be composed of 91.30% OC and 8.7% TC. Among these, significant metabolites were limonene (37.33%), decanal (18.24%), and *α*-pinene (15.74%). The flavor of HP comprised 68.49% OC and 31.51% TC, with significant metabolites being *γ*-muurolene (27.61%) and citric acid (15.09%). The flavor of KP comprised 31.22% OC and 68.78% TC, with significant metabolites being citric acid (59.89%) and murolene (19.47%).

OC and TC results indicate that key flavor metabolites contributing to the flavor of CFPs were identified as decanal, *α*-pinene, limonene, 2-carene, *γ*-muurolene, and citric acid, exhibiting a similar pattern to previous studies (Jo et al., 2025; Kowalska et al., 2023; Liang et al., 2022). The flavor profiles of OP, MP, LP, CP, RP, and HP indirectly indicate that they are more influenced by metabolites associated with odor than those with taste. Among these, limonene and 2-carene exhibited higher proportions than other flavor metabolites and are known odor compounds abundant in the essential oil of CFP and the plant itself (Weng et al., 2024). By contrast, the flavor profile of KP appeared to be more strongly influenced by taste-related metabolites than by OACs. Notably, citric acid—an OA contributing to sour taste—accounted for the highest proportion at 59.89% (Wang, Xie, et al., 2024). The overall flavor of CP and RP exhibited similar patterns, and these results may be related to a similar combination (limonene, decanal, and *α*-pinene) of OACs. Similarly, the overall flavor of HP and KP exhibited similar patterns due to a similar combination (*γ*-muurolene and citric acid) of OACs. TAV/OAV-based indices provide a pragmatic estimate of potential taste/odor contribution by relating concentration to reported threshold values; however, they do not reflect perceived intensity in complex food matrices (Grosch, 2001). Human perception is influenced by interactions among mixtures, matrix effects, and cross-modal odor–taste integration (Peris & Escuder-Gilabert, 2016). Therefore, relationships between chemical markers and *E*-tongue/E-nose outputs in this study should be interpreted as correlations rather than direct cause–effect evidence.

### 3.6 Multivariate analysis

**Fig. 6:**
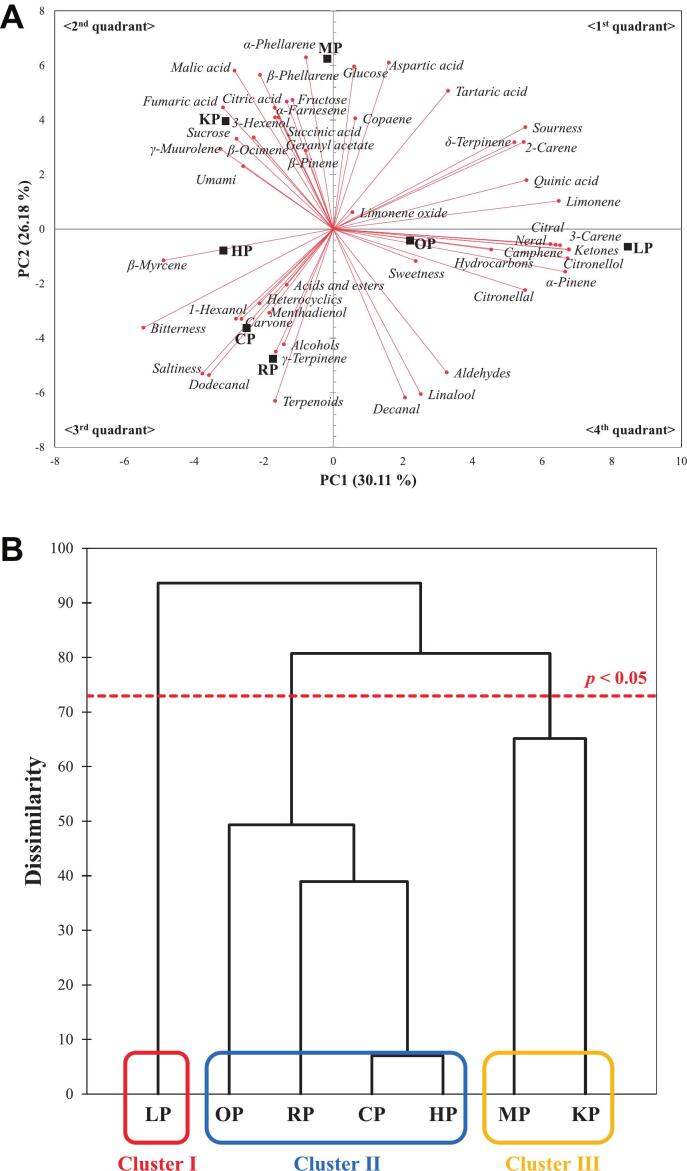

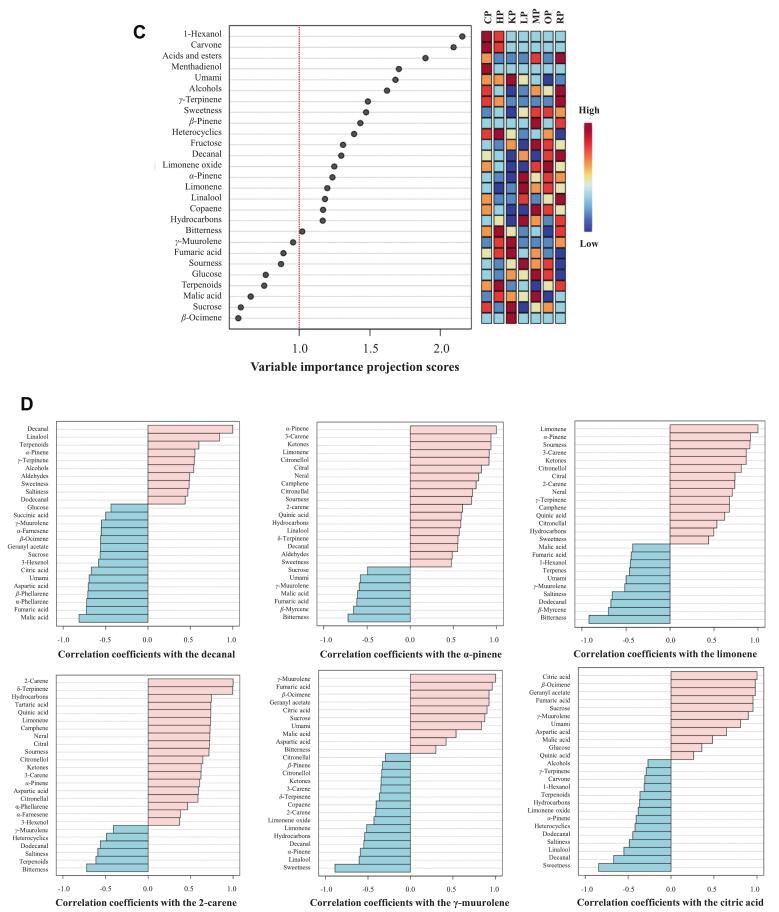

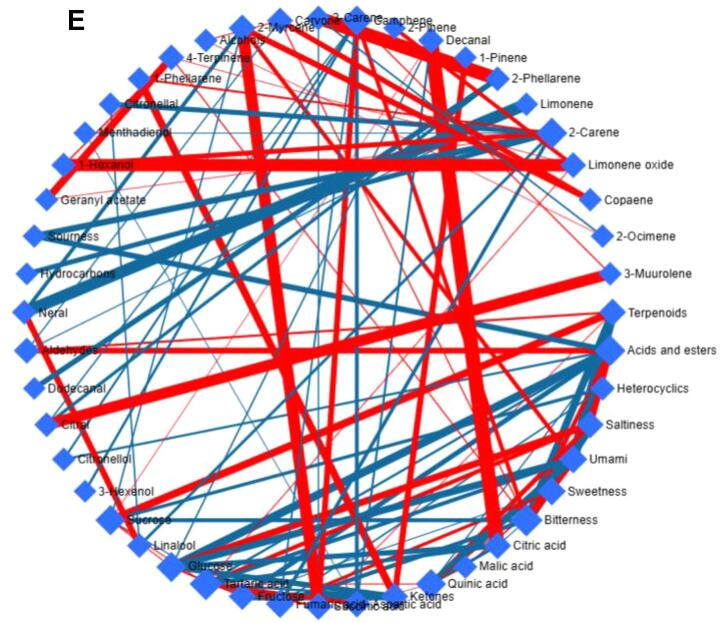
(A) Principal component analysis (PCA), (B) hierarchical cluster analysis (HCA), (C) partial least squares discriminant analysis (PLS-DA), (D) pattern hunter analysis, and (E) debiased sparse partial correlation (DSPC) network of flavor metabolite data from citrus fruit peels (CFPs). OP: orange peel, MP: mandarin orange peel, LP: lemon peel, CP: cheonhyehyang peel, RP: redhyang peel, HP: hallabong peel, and KP: kumquat peel. (For interpretation of the references to colour in this figure legend, the reader is referred to the web version of this article.)

To classify flavor metabolites of CFPs and their correlations, PCA, HCA, PLS-DA, pattern hunter analysis, and DSPC network were applied. The PCA and HCA results for CFPs are shown in Fig. 6A and B, respectively. PC1 accounted for 26.18% of the variance, while PC2 explained 30.11%, yielding a cumulative variance of 56.29%. MP and KP were located in the second quadrant. These showed higher correlations with NMS, *γ*-muurolene, *β*-ocimene, geranyl acetate, *α-*phellarene, *β*-phellarene, *α*-farnesene, 3-hexenol, *β*-pinene, succinic acid, fumaric acid, citric acid, malic acid, fructose, and sucrose among flavor compounds compared to other CFPs (*p* < 0.05). In the third quadrant, HP, CP, and RP were located, and they showed higher correlations with flavor compounds such as SCS, CTS, *β*-myrcene, 1-hexanol, dodecanal, carvone, heterocyclics, acids and esters, alcohols, *γ*-terpinene, terpenoids, and menthadienol compared to other CFPs (*p* < 0.05). The fourth quadrant contained OP and LP, which showed higher correlations with ANS, citronellal, *α*-pinene, hydrocarbons, camphene, neral, citral, ketones, 3-carene, and citronellol compared to other CFPs (*p* < 0.05). HCA results demonstrated the formation of three clusters (Fig. 6B). Cluster I comprised LP, cluster II comprised OP, RP, CP, and HP, and cluster III comprised MP and KP. The separation between cluster I and cluster II is presumed to result from the elevated levels of terpenes and quinic acid in LP. By contrast, the distinction between cluster II and cluster III was attributed to the rich presence of OAs and FSs in KP and MP. These compositional differences indicate that the high terpene and quinic acid content in LP influences its clustering, while KP and MP are more strongly characterized by taste-related metabolites.

Differences in flavor metabolites of CFPs were investigated using PLS-DA. PLS-DA was applied to identify key factors (variable importance projection; VIP) for classification. Flavor metabolites (VIP scores ≥1) were considered crucial for sample classification (Hong et al., 2025; Oussama et al., 2012). Fig. 6C presents the top 18 flavor metabolites, namely, 1-hexanol (2.20), carvone (2.13), acids and esters (1.93), menthadienol (1.74), NMS (1.71), alcohols (1.65), *γ*-terpinene (1.52), ANS (1.50), *β*-pinene (1.46), heterocyclics (1.42), fructose (1.33), decanal (1.32), limonene oxide (1.27), *α*-pinene (1.26), limonene (1.22), linalool (1.20), copaene (1.19), hydrocarbons (1.18), and bitterness (1.04).

The results of the pattern hunter analysis are shown in Fig. 6D. The pattern hunter analysis is visualized based on Pearson r distance, allowing individual monitoring of the selected metabolite in its interaction with other metabolites. Results are illustrated as positive correlation (red) and negative correlation (blue) (Ban et al., 2025). This study focuses on identifying correlations between key flavor metabolites (TC or OC ≥ 10%)—recognized as primary contributors to the flavor of CFPs—and other associated metabolites (Jo et al., 2025; Kowalska et al., 2023; Liang et al., 2022). Decanal showed a positive correlation with linalool (0.84) (*p* < 0.05) and a negative correlation with malic acid (−0.82) (*p* < 0.05). Similarly, *α*-pinene was positively correlated with limonene (0.92) and sourness (0.71) (*p* < 0.05) and negatively correlated with malic acid (−0.62) (*p* < 0.05). Furthermore, limonene exhibited a positive correlation with sourness (0.91) (*p* < 0.05) and a negative correlation with SCS (−0.92) (*p* < 0.05). 2-Carene exhibited positive correlations with limonene (0.74) and sourness (0.72) (*p* < 0.05) and a negative correlation with SCS (−0.72) (*p* < 0.05). Similarly, *γ*-muurolene was positively correlated with fumaric acid (0.96) (*p* < 0.05) and negatively correlated with limonene (−0.51) and ANS (−0.89) (*p* < 0.05). Citric acid exhibited a positive correlation with geranyl acetate (0.98) (*p* < 0.05) and a negative correlation with ANS (−0.84) (*p* < 0.05). OP, MP, LP, CP, and RP contained abundant flavor metabolites—*α*-pinene, limonene, and 2-carene—which were positively correlated with sourness (0.71, 0.91, and 0.72, respectively) and negatively correlated with SCS (−0.72, −0.92, and − 0.72, respectively). By contrast, HP and KP, characterized by high levels of *γ*-myrcene and citric acid, showed negative correlations with ANS (−0.89 and − 0.84, respectively). These results indicate that an increase in primary flavor metabolites of CFPs is associated with increasing sourness and decreased ANS and SCS. These results indicate that sweetness perception in complex foods is significantly shaped by mixture interactions, and that the sweetness-sourness inhibition phenomenon is well known as the contribution of OAs increases, perceived sweetness in the sucrose–acid relationship may weaken (Green et al., 2010; Hewson et al., 2008). Therefore, the negative correlations observed in this study should be understood not as a direct sweetness-inhibiting mechanism of odor compounds themselves, but rather as potentially reflecting varietal-level co-variation (the phenomenon in which terpene-rich peel occurs alongside an acid-dominant matrix). Similar to the results of the pattern hunter analysis in this study, Ashby and Townsend (1986) reported identifying a close connection between citrus flavor and sour taste.

The results of the DSPC network are presented in Fig. 6E, which was represented using the DSPC algorithm based on the declassified graphical lasso modeling procedure. This technique is widely used in research on the food industry to understand the correlation between metabolites and samples (Jo et al., 2025). The DSPC network comprises nodes representing metabolites, along with their degree and betweenness centrality (Table S3). Edges connecting the nodes are colour-coded: red indicates positive correlations, while blue denotes negative correlations. “Degree” refers to the number of connections a node has with other nodes, and degree signifies the number of connections between them. Therefore, metabolites with high degree and betweenness centrality are considered essential within the flavor network (Marino et al., 2022; Tian et al., 2024). SCS, with a degree of 11 and a betweenness value of 214.27, was identified as the most influential metabolite in the DSPC network. Previous studies indicate that SCS is a typical flavor characteristic of CFs, varying with fruit type, anatomical part, and growth conditions (Rahman et al., 2024). Therefore, the observed results are considered to be partially associated with the DSPC network.

A limitation of this study is that no trained human sensory panel test was conducted; thus, the link between electronic sensor (*E*-tongue and E-nose) outputs and human perception cannot be directly established. While electronic sensors provide objective, repeatable pattern-based fingerprints, sensory validation and mixture reconstitution/omission tests are required to establish perceptual causality.

## 4 Conclusion

This study profiled seven citrus fruit peels (CFPs) using an integrated workflow combining biomimetic sensing (E-tongue/E-nose) with targeted analyses of taste and odor compounds (LC–MS/MS and GC–O–MS). Cross-platform integration with contribution indices (TAV/OAV and TC/OC) and multivariate modeling revealed cultivar-dependent fingerprints and classified CFPs into three clusters characterized by distinct acid–sugar matrices and a limited set of key odorants; for example, citric acid peaked in kumquat peel (2152.38 ± 18.84 mg/kg), highlighting the cultivar-dependent divergence in taste-active composition.

From an application-oriented screening perspective, these cluster-level fingerprints suggest candidate CFP groups for different formulation targets. The quinic-acid–associated, terpene-forward cluster (Cluster I; LP) may be considered for applications aiming for bright acidity and fresh citrus top notes, whereas the odorant-rich cluster (Cluster II; OP, RP, CP, HP), featuring representative citrus odorants (e.g., limonene, α-pinene, and decanal), may be prioritized for aroma enrichment in citrus-flavored matrices. The acid–sugar–enriched cluster (Cluster III; MP, KP) may serve as a candidate for products targeting a balanced sweet–sour profile. In practice, the proposed workflow can serve as a rapid screening strategy to prioritize citrus peel resources for different product concepts (e.g., acid-forward formulations, aroma-enrichment ingredients, or balanced profiles) before conducting time- and cost-intensive sensory testing. We emphasize that the multivariate associations reported here represent correlation-based fingerprints. Importantly, metabolite–sensor relationships and threshold-based contribution indices reflect correlation- and model-based inferences. Perceived flavor in foods can be modulated by matrix effects and mixture/cross-modal interactions. Therefore, this study had a few limitations, including the need for sensory-panel validation in further studies to confirm perception-level relevance and to translate candidate markers into robust formulation guidance. Future studies combining receptor-focused assays with human sensory validation may further strengthen the mechanistic understanding of the key candidate markers identified here.

## CRediT authorship contribution statement

**Hee Sung Moon:** Writing – original draft, Methodology, Conceptualization. **Seong Jun Hong:** Writing – original draft, Methodology, Conceptualization. **Se Young Yu:** Methodology, Conceptualization. **Hyeonjin Park:** Methodology, Formal analysis. **Younglan Ban:** Methodology, Formal analysis. **Gwang-Ju Jang:** Methodology, Formal analysis. **Ho Eun Kim:** Methodology, Formal analysis. **Sang-Hee Lee:** Writing – review & editing, Methodology, Funding acquisition, Conceptualization. **Eui-Cheol Shin:** Writing – review & editing, Supervision, Methodology, Conceptualization.

## Funding

This study was supported by grants from the Korea Food Research Institute (E0232203).

## Declaration of competing interest

The authors declare that they have no known competing financial interests or personal relationships that could have appeared to influence the work reported in this paper.

## Data Availability

Data will be made available on request.
