# Supplementary material for: Elucidation of flavor codes in citrus fruit peels using electronic sensors, LC-MS/MS, and GC–MS combined with GC-O
Source: Food Chem X. 2026 Jun 6;37:104074. doi: 10.1016/j.fochx.2026.104074 (PMC13262289; doi:10.1016/j.fochx.2026.104074)
Supplement: Supplementary file 1 — Supplementary material [file mmc1.docx]

**Table S1.** Free sugar (FS) and organic acid (OA) contents analyzed by LC-MS/MS and GC-MS.

|  | **FS (mg/g)** | | |  | **OA (mg/kg)** | | | | | | |
| --- | --- | --- | --- | --- | --- | --- | --- | --- | --- | --- | --- |
| **Samples** | **Fructose** | **Glucose** | **Sucrose** |  | **Citric acid** | **Malic acid** | **Quinic acid** | **Aspartic acid** | **Succinic acid** | **Fumaric acid** | **Tartaric acid** |
| **OP** | 22.64±0.40^b^ | 22.54±0.67^a^ | 2.81±0.12^bc^ |  | 73.16±0.75^e^ | 46.30±1.02^f^ | 193.35±2.26^d^ | 12.58±0.18^e^ | 0.08±0.00^b^ | 0.86±0.03^e^ | 1.14±0.01^c^ |
| **MP** | 25.68±0.60^a^ | 24.11±0.62^a^ | 2.59±1.19^bc^ |  | 418.88±21.62^b^ | 1501.55±23.88^a^ | 317.75±8.02^c^ | 73.88±2.04^a^ | 15.90±5.34^a^ | 3.83±0.13^c^ | 1.56±0.02^a^ |
| **LP** | 8.16±0.09^f^ | 11.10±0.46^c^ | 0.40±0.02^e^ |  | 299.50±10.61^c^ | 227.98±6.36^d^ | 1018.75±15.90^a^ | 60.25±0.67^b^ | 0.20±0.03^b^ | 0.91±0.04^e^ | 1.50±0.05^a^ |
| **CP** | 11.90±0.52^e^ | 10.37±0.35^cd^ | 3.65±0.07^b^ |  | 22.42±0.90^f^ | 95.91±4.87^ef^ | 190.17±4.95^d^ | 1.62±0.25^f^ | 0.13±0.10^b^ | 1.39±0.06^d^ | 0.74±0.05^d^ |
| **RP** | 14.20±0.24^d^ | 7.51±0.08^e^ | 1.87±0.32^cd^ |  | 20.54±0.89^f^ | 172.85±4.80^de^ | 105.37±2.48^e^ | 24.19±0.19^d^ | 0.19±0.05^b^ | 0.78±0.02^e^ | 0.74±0.01^d^ |
| **HP** | 10.69±2.29^e^ | 8.41±1.82^de^ | 0.82±0.16^de^ |  | 152.32±7.23^d^ | 1305.65±85.58^b^ | 187.84±0.95^d^ | 27.62±1.16^c^ | 12.53±0.71^a^ | 4.65±0.23^b^ | 1.40±0.02^b^ |
| **KP** | 17.21±0.93^c^ | 19.01±1.03^b^ | 18.48±0.19^a^ |  | 2152.38±18.84^a^ | 1198.67±8.21^c^ | 468.17±13.94^b^ | 71.85±1.36^a^ | 0.16±0.01^b^ | 13.69±0.27^a^ | 1.09±0.04^c^ |

Different letters (a–f) indicate significant differences, as determined by Tukey's multiple range test (*p* < 0.05).

The abbreviations for all CFPs are as follows: OP, orange peel; MP, mandarin orange peel; LP, lemon peel; CP, cheonhyehyang peel; RP, redhyang peel; HP, hallabong peel; KP, kumquat peel.

**Table S2.** Odor-active value (OAV), Taste-active value (TAV), odor contribution (OC), and taste contribution (TC) of flavor metabolites.

| Flavor metabolites | Thresholds (odor or taste) | OAV · TAV | | | | | | |  | OC · TC (%) | | | | | | | References |
| --- | --- | --- | --- | --- | --- | --- | --- | --- | --- | --- | --- | --- | --- | --- | --- | --- | --- |
|  |  | OP | MP | LP | CP | RP | HP | KP |  | OP | MP | LP | CP | RP | HP | KP |  |
| **OACs** |  |  |  |  |  |  |  |  |  |  |  |  |  |  |  |  |  |
| Geranyl acetate | 150 µg/kg |  | - | - | - | - | - | < 1 |  | - | - | - | - | - | - | - | Qi et al., 2020 |
| 3-Hexenol | 4 µg/kg | - | 4.76 | - | 1.63 | - | 1.6 | - |  | - | 0.57 | - | 0.77 | - | 0.68 | - | Czerny et al., 2008 |
| 1-Hexanol | 110 µg/kg | - | - | - | < 1 | - | < 1 | - |  | - | - | - | - | - | - | - | Arcari et al., 2017 |
| Linalool | 28 µg/kg | 5.84 | - | 6.32 | 6.13 | 9.33 | - | - |  | 1.14 | - | 0.53 | 2.88 | 3.67 | - | - | Jo et al., 2025 |
| Citronellol | 42 µg/kg | 5.06 | - | 13.55 | < 1 | < 1 | < 1 | - |  | 0.99 | - | 1.14 | - | - | - | - | Peltz, 2015 |
| Citronellal | 30 µg/kg | - | - | 7.82 | - | 3.11 | < 1 | - |  | - | - | 0.66 | - | 1.22 | - | - | Qi et al., 2020 |
| Decanal | 3 µg/kg | 43.95 | - | 29.58 | 23.17 | 46.36 | 22.47 | - |  | 8.61 | - | 2.49 | 10.87 | 18.24 | 9.58 | - | Qi et al., 2020 |
| Neral | 200 µg/kg | < 1 | - | 2.33 | - | - | - | - |  | - | - | 0.20 | - | - | - | - | Villavicencio et al., 2021 |
| Citral | 32 µg/kg | 2.86 | - | 24.07 | - | - | - | - |  | 0.56 | - | 2.02 | - | - | - | - | Qi et al., 2020 |
| Dodecanal | 10 µg/kg | - | - | - | 1.15 | 2.15 | 1.73 | - |  | - | - | - | 0.54 | 0.85 | 0.74 | - | Qi et al., 2020 |
| *α*-Pinene | 14 µg/kg | 76.99 | 29.18 | 106.15 | 21.65 | 40.02 | 9.51 | 2.68 |  | 15.08 | 3.48 | 8.92 | 10.16 | 15.74 | 4.05 | 0.32 | Qi et al., 2020 |
| Camphene | 130 µg/kg | - | - | 3.29 | - | - | - | - |  | - | - | 0.28 | - | - | - | - | Czerny et al., 2008 |
| *β*-Pinene | 140 µg/kg | - | 2.57 | - | - | 1.43 | - | - |  | - | 0.31 | - | - | 0.56 | - | - | Qi et al., 2020 |
| *β*-Myrcene | 28 µg/kg | - | 16.8 | - | 14.17 | 22.79 | 14.07 | 8.8 |  | - | 2.00 | - | 6.65 | 8.97 | 6.00 | 1.05 | Peltz, 2015 |
| *β*-Phellandrene | 5 µg/kg | - | 12.63 | - | - | - | 7.53 | 3.73 |  | - | 1.51 | - | - | - | 3.21 | 0.45 | Qi et al., 2020 |
| *α*-Phellandrene | 500 µg/kg | - | < 1 | - | - | - | - | < 1 |  | - | - | - | - | - | - | - | Czerny et al., 2008 |
| Limonene | 34 µg/kg | 255.17 | 182.61 | 308.56 | 93.98 | 94.88 | 14.27 | 77.29 |  | 49.97 | 21.78 | 25.94 | 44.11 | 37.33 | 6.08 | 9.25 | Jo et al., 2025 |
| 3-Carene | 1200 µg/kg | 1.73 | - | 3.43 | < 1 | - | - | - |  | 0.34 | - | 0.29 | - | - | - | - | Czerny et al., 2008 |
| *γ*-Terpinene | 1000 µg/kg | - | - | - | < 1 | 1.17 | < 1 | - |  | - | - | - | - | 0.46 | - | - | Qi et al., 2020 |
| 2-Carene | 5 µg/kg | 68.1 | 381.8 | 464.79 | 20.16 | 5.18 | 17.91 | 5.77 |  | 13.34 | 45.55 | 39.07 | 9.46 | 2.04 | 7.63 | 0.69 | Czerny et al., 2008 |
| *δ*-Terpinene | 10 µg/kg | - | 31.72 | 39.1 | - | - | - | - |  | - | 3.78 | 3.29 | - | - | - | - | Czerny et al., 2008 |
| Limonene oxide | 80 µg/kg | 2.56 | 1.19 | - | < 1 | < 1 | < 1 | - |  | 0.50 | 0.14 | - | - | - | - | - | Czerny et al., 2008 |
| Carvone | 160 µg/kg | - | - | - | < 1 | - | < 1 | - |  | - | - | - | - | - | - | - | Qi et al., 2020 |
| Copaene | 95 µg/kg | < 1 | < 1 | - | < 1 | < 1 | < 1 | - |  | - | - | - | - | - | - | - | Czerny et al., 2008 |
| *β*-Ocimene | 34 µg/kg | - | - | - | - | - | - | < 1 |  | - | - | - | - | - | - | - | Qi et al., 2020 |
| *γ*-Muurolene | 0.25 µg/kg | - | - | - | - | 5.68 | 64.8 | 162.72 |  | - | - | - | - | 2.23 | 27.61 | 19.47 | Asikin et al., 2024 |
| *α*-Farnesene | 18 µg/kg | - | 13.07 | - | < 1 | - | 6.82 | - |  | - | 1.56 | - | - | - | 2.91 | 0.00 | Czerny et al., 2008 |
|  |  |  |  |  |  |  |  |  |  |  |  |  |  |  |  |  |  |
| **FSs** |  |  |  |  |  |  |  |  |  |  |  |  |  |  |  |  |  |
| Fructose | 8.32 mg/g | 2.72 | 3.09 | < 1 | 1.43 | 1.71 | 1.28 | 2.07 |  | 0.53 | 0.37 | - | 0.67 | 0.67 | 0.55 | 0.25 | Pasquet et al., 2006 |
| Glucose | 2.43 mg/g | 9.28 | 9.92 | 4.57 | 4.27 | 3.09 | 3.46 | 7.82 |  | 1.82 | 1.18 | 0.38 | 2.00 | 1.22 | 1.47 | 0.94 | Breslin et al., 2021 |
| Sucrose | 13.73 mg/g | < 1 | < 1 | < 1 | < 1 | < 1 | < 1 | 1.35 |  | - | - | - | - | - | - | 0.16 | Pasquet et al., 2006 |
|  |  |  |  |  |  |  |  |  |  |  |  |  |  |  |  |  |  |
| **OAs** |  |  |  |  |  |  |  |  |  |  |  |  |  |  |  |  |  |
| Citric acid | 4.3 mg/kg | 17.01 | 97.41 | 69.65 | 5.21 | 4.78 | 35.42 | 500.55 |  | 3.33 | 11.62 | 5.85 | 2.45 | 1.88 | 15.09 | 59.89 | Li & Liu, 2015 |
| Malic acid | 87 mg/kg | < 1 | 17.26 | 2.62 | 1.10 | 1.99 | 15.01 | 13.78 |  | - | 2.06 | 0.22 | 0.52 | 0.78 | 6.40 | 1.65 | Chen et al., 2023 |
| Quinic acid | 10 mg/kg | 19.34 | 31.78 | 101.88 | 19.02 | 10.54 | 18.78 | 46.82 |  | 3.79 | 3.79 | 8.56 | 8.93 | 4.15 | 8.00 | 5.60 | Lin et al., 2022 |
| Aspartic acid | 30 mg/kg | < 1 | 2.46 | 2.01 | < 1 | < 1 | < 1 | 2.40 |  | - | 0.29 | 0.17 | - | - | - | 0.29 | Kang et al., 2023 |
| Succinic acid | 22 mg/kg | < 1 | < 1 | < 1 | < 1 | < 1 | < 1 | < 1 |  | - | - | - | - | - | - | - | Li & Liu, 2015 |
| Fumaric acid | 38.6 mg/kg | < 1 | < 1 | < 1 | < 1 | < 1 | < 1 | < 1 |  | - | - | - | - | - | - | - | Li & Liu, 2015 |
| Tartaric acid | 6 mg/kg | < 1 | < 1 | < 1 | < 1 | < 1 | < 1 | < 1 |  | - | - | - | - | - | - | - | Li & Liu, 2015 |

Note: The thresholds for calculating OAV and TAV were found in the following references [Arcari et al., 2017; Asikin et al., 2024; Breslin et al., 2021; Chen et al., 2023; Czerny et al., 2008; Jo et al., 2025; Kang et al., 2023; Li & Liu, 2015; Lin et al., 2022; Pasquet et al., 2006; Peltz, 2015; Qi et al., 2020; Villavicencio et al., 2021].

The abbreviations for all CFPs are as follows: OP, orange peel; MP, mandarin orange peel; LP, lemon peel; CP, cheonhyehyang peel; RP, redhyang peel; HP, hallabong peel; KP, kumquat

**Table S3.** Correlation network analysis. Degree of node and betweenness of the interrelationships calculated on the flavor metabolites with VIP score ≥ 1 related to taste patterns, volatile compounds, and OACs of citrus fruit peels.

| ***No.*** | **Flavor metabolites** | **Degree** | **Betweenness** |
| --- | --- | --- | --- |
| *1* | Bitterness | 11 | 214.27 |
| *2* | Tartaric acid | 11 | 195.63 |
| *3* | 2-Carene | 9 | 186.78 |
| *4* | Acids and esters | 9 | 111.24 |
| *5* | Camphene | 8 | 202.66 |
| *6* | Sucrose | 8 | 64.85 |
| *7* | Quinic acid | 8 | 41.42 |
| *8* | Glucose | 8 | 32.76 |
| *9* | Fumaric acid | 6 | 135.39 |
| *10* | Sweetness | 6 | 34.82 |
| *11* | Umami | 6 | 31.59 |
| *12* | Succinic acid | 5 | 131.8 |
| *13* | 2-Myrcene | 5 | 74.97 |
| *14* | Ketones | 5 | 72.93 |
| *15* | Aspartic acid | 5 | 56.13 |
| *16* | Terpenoids | 5 | 26.4 |
| *17* | Fructose | 5 | 17.37 |
| *18* | Citric acid | 5 | 11.06 |
| *19* | Saltiness | 5 | 5.24 |
| *20* | Carvone | 4 | 75.57 |
| *21* | Limonene oxide | 4 | 62.86 |
| *22* | 4-Terpinene | 4 | 46.48 |
| *23* | Aldehydes | 4 | 44.11 |
| *24* | Decanal | 4 | 6.61 |
| *25* | 3-Carene | 3 | 71.72 |
| *26* | 2-Phellarene | 3 | 49.5 |
| *27* | Citral | 3 | 47.49 |
| *28* | Neral | 3 | 35.11 |
| *29* | Linalool | 3 | 25.92 |
| *30* | 1-Hexanol | 3 | 15.08 |
| *31* | Heterocyclics | 3 | 2.71 |
| *32* | Sourness | 2 | 17.58 |
| *33* | Alcohols | 2 | 16.53 |
| *34* | 1-Phellarene | 2 | 16.38 |
| *35* | Menthadienol | 2 | 11.53 |
| *36* | Citronellol | 2 | 7.66 |
| *37* | 2-Ocimene | 2 | 6.74 |
| *38* | Limonene | 2 | 6.43 |
| *39* | Copaene | 2 | 4.12 |
| *40* | Geranyl acetate | 2 | 3.58 |
| *41* | Malic acid | 2 | 0 |
| *42* | Citronellal | 2 | 0 |
| *43* | 3-Muurolene | 2 | 0 |
| *44* | 3-Hexenol | 1 | 0 |
| *45* | Dodecanal | 1 | 0 |
| *46* | Hydrocarbons | 1 | 0 |
| *47* | 2-Pinene | 1 | 0 |
| *48* | 1-Pinene | 1 | 0 |
